# Stretching after spinal cord injury: a call for evidence for this common clinical practice

**DOI:** 10.3389/fresc.2024.1505439

**Published:** 2024-12-10

**Authors:** Todd E. Williams, Louis A. DeMark, Tinuade A. Olarewaju, Kelly A. Hawkins, Emily J. Fox

**Affiliations:** ^1^ Department of Physical Therapy, University of Florida, Gainesville, FL, United States; ^2^ Brooks Rehabilitation, Clinical Research and Motion Analysis Centers, Jacksonville, FL, United States

**Keywords:** stretching, rehabilitation, motor output, spasticity, walking function, spinal cord injury

## Abstract

Stretching is a ubiquitous rehabilitation intervention for individuals with spinal cord injury (SCI), intended to reduce spasticity, maintain or improve joint range of motion, and prevent joint contractures. Although people with SCI report that stretching is their preferred approach to reduce spasticity, limited evidence supports the use of stretching for people with SCI, including short-term (< one hour) effects on spasticity. Further, the long-term effects and the effects of stretching on motor function have yet to be examined in humans with SCI. Evidence from pre-clinical studies in rats with SCI demonstrates that stretching impairs motor output, reduces spinal cord excitability, and abolishes walking function. This perspective paper discusses evidence of static stretching in humans and rats with SCI regarding the effects on range of motion, joint contractures, and effects on voluntary and involuntary (i.e., spasticity) motor output. Additionally, we aim to challenge assumptions regarding the use of stretching and encourage research to advance the understanding of this common rehabilitation approach. Research is needed to investigate underlying mechanisms of stretch-induced effects and to advance stretching protocols to optimize the potential beneficial effects of stretching for people with SCI.

## 1 Introduction

The purpose of this perspective paper is to discuss evidence regarding the effects of stretching as a rehabilitation strategy for people with spinal cord injury (SCI). After SCI, static stretching is applied to achieve muscle and soft tissue elongation by maintaining a joint or body position for∼20–60 s (1). Stretching is assumed to have clinical benefits and is utilized by nearly all (∼95%) patients with SCI (2–4) and is completed regularly (i.e., 3–5 times per week) in all clinical settings, including in-home exercise programs (4, 5). Despite the ubiquitous application of stretching in people with SCI, the evidence to support its efficacy is lacking.

Stretching is commonly prescribed to maintain joint range of motion (ROM) and prevent joint contractures. However, studies examining the effects of stretching in people with SCI have concluded that stretching does not have clinically meaningful effects on joint mobility (2). Stretching is also used to reduce spasticity, a complex condition characterized by spasms and involuntary motor activity experienced by more than 50% of individuals with SCI (6). However, to our knowledge, only a single study has reported reductions in spasticity following a single episode of stretching (7). Additionally, accumulating evidence in SCI rodent models indicates that stretching impairs motor output and diminishes locomotor function (8, 9).

With the extensive use of stretching in standard SCI rehabilitation, examining the evidence supporting this practice is essential. Therefore, this perspective paper discusses evidence in humans and rats with SCI concerning the effects of static stretching on range of motion and contractures and the impact on voluntary and involuntary motor output. Furthermore, it aims to challenge assumptions regarding the use of stretching and to encourage research and the development of guidelines for the use of stretching.

## 1.1 The effects of stretching on joint ROM and contractures

### 1.1.1 Stretching does not have a clinically meaningful effect on ROM or reduce the risk for contractures

Contractures are common in people with SCI, interfering with function and negatively impacting quality of life (10, 11). Following SCI, stretching is often applied to mitigate the risk of contractures and maintain range of motion (10). Despite its ubiquitous use and integration into rehabilitative care, little evidence supports the use of stretching to affect joint ROM or reduce the risk of contractures (4, 10).

A meta-analysis concluded that multiple forms of stretching typically performed in the clinic do not have clinically meaningful effects on joint mobility for people at risk for contractures, including people with SCI (2). The review broadly evaluated the application of stretching, including its use in those with musculoskeletal conditions. It also analyzed a subgroup of studies focused on people with neurologic conditions, including those with SCI. Across all patient conditions and subgroup analyses, stretching did not have a clinically meaningful effect on joint mobility. Specifically, for those with SCI, stretching for five days per week over a thirty-minute duration for four weeks resulted in no improvements to ankle or knee range of motion (12, 13).

Limited research on the effects of stretching has been conducted in experimental animals with SCI (14, 15). These studies suggest that further research is needed to determine if stretching can prevent and improve ROM. Moriyama and colleagues developed a protocol for stretching where distraction was used with variable amounts of torque to the hindlimbs of rats with SCI (15). Using this protocol, they found that the highest torque level applied improved joint ROM (15). However, these high levels of torque and use of distraction are different than static stretching applied in rehabilitation, making it unlikely to translate to clinical protocols. Conversely, Caudle and colleagues developed a stretching protocol modeled after common rehabilitation strategies (i.e., comparable torques and time of stretching bouts to what is provided in humans). They found that stretching did not reduce the development of contractures (14). The protocol, initiated four days post-injury and applied five days per week, involved two sets of one-minute stretches applied to six muscles of each hindlimb (∼24–30 min of stretching). Surprisingly, not only did stretching not prevent contractures, but dramatic impairments in hindlimb and locomotor function were evident (14). The results from these two studies indicate that further research is needed to test the effects of stretching and develop new stretching protocols that may be clinically feasible and effective for improving ROM and reducing contractures after SCI.

## 2 The effects of stretching on involuntary motor output

### 2.1 stretching diminishes spinal excitability, alters motor activation, and may reduce spasticity

Approximately 65%–78% of individuals with chronic SCI (greater than one year post-injury) experience spasticity, characterized by involuntary muscle activity and over-responsive reflexes leading to resistance to passive stretch (16). Spasticity typically includes frequent spasms that negatively impacts self-care, daily functioning, and walking (6, 17). Effective interventions for spasticity are limited, with standard treatments, like baclofen and other antispasmodic medications, lacking sufficient supportive evidence and often causing unwanted side effects (18–22).

Limited empirical evidence supports the use of stretching for spasticity in people with SCI (23). Although individuals with SCI report stretching as their preferred method of spasticity management, their preferences regarding influential stretching variables (type, duration, and location) have not been reported, limiting understanding of how stretching is typically used by those who report benefit (6). Estes et al. studied ten individuals with chronic SCI. They found that a single session of stretching, comprising three sets of five bilateral lower extremity static stretches lasting one minute each (approximately thirty minutes of stretching), led to short-term reductions in quadriceps muscle spasticity, as assessed by the pendulum test—a standardized measure of quadriceps spasticity (7). Notably, these reductions in spasticity were no longer statistically significant forty-five minutes after stretching (7). Further evidence is needed to determine if stretching has a durable effect on spasticity and its impacts on function and quality of life. Additionally, protocols for stretching need further examination and optimization to maximize the benefits for those seeking to reduce spasticity.

Stretching may reduce spasticity by inhibiting spinal reflexes (24). In animal models with chronic SCI, ten weeks post-injury, stretched rats demonstrated a reduction in magnetically evoked tail reflex amplitudes (25). Tail reflexes, which are thought to be similar to the Hoffman monosynaptic reflex, were recorded from bilateral gastrocnemius muscles in stretched and un-stretched rats. The stretch group's reflexes were reduced and diminished for weeks after stretching ceased, suggesting that stretching induced a persistent decrease in motoneuron or spinal circuit excitability (25). Changes in electromyography on the unstretched, contralateral limb of the rats, following the stretching protocol, suggest that stretching affects central neural circuitry (vs. peripheral or local/limb specific effects) (26). While limited evidence supports the potential short-term benefits of stretching on spasticity, altering the excitability of spinal circuits raises the possibility of reduced motor output following stretching.

## 3 The effects of stretching on voluntary motor output

### 3.1 stretching abolishes locomotion and hindlimb function in rat models of SCI contusion injuries

Voluntary motor output, such as during walking or voluntary muscle contractions, is often diminished or absent following SCI. Unfortunately, to our knowledge, no studies evaluate the effect of stretching on voluntary motor output in humans with SCI. However, several studies using rodent models of SCI evaluated stretching-induced effects on locomotion and hindlimb voluntary function.

In a series of studies conducted in chronic spinal cord contusion injured rats, Caudle and colleagues demonstrated that stretching abolished locomotion and hind limb function (8, 14). Stretching was applied daily, five days per week, for eight weeks. The assessments were performed three times a week, before and after a single stretching session, on the first day of the week, and then after the last stretching session on the last day of the week. Study outcomes indicate that stretch-induced effects accumulate across daily sessions, evidenced by the extreme locomotor decrement on day five of the weekly sessions. After two days without stretching (i.e., after the weekend), the effects disappeared, and function returned to baseline (8, 14).

Subsequent studies reproduced initial findings in acutely injured rats and compared them to rats with chronic contusion injuries (ten weeks post-injury with stable locomotor function) (8, 27). The chronically injured rats exhibited significantly greater declines in locomotor function than the acutely injured rats (27). After five daily stretches, these rats showed substantial functional declines, characterized by reduced hindlimb joint movements and dragging of the hindlimbs (27). Although their function improved four weeks after discontinuing the stretching, the results suggest that chronically injured animals are particularly vulnerable to the negative impacts of daily stretching (27).

The presence of nociceptor afferents seems to be needed to observe the stretch-induced effects on voluntary motor output (9). This is evident from studies of rats with SCI that demonstrate animals with intact nociceptors [transient receptor potential cation channel subfamily V member 1 (TRPV1+)] display motor impairments in response to stretching. In contrast, animals who received capsaicin injections as neonates, and therefore had depleted nociceptors, did not demonstrate such impairments. Animals with motor impairments also had increased dorsal horn calcitonin gene-related peptide (CGRP) levels, a marker for nociceptive C fiber primary afferents (9). This suggests that stretching may encourage intra-spinal sprouting of these fibers, which is linked to adverse SCI conditions such as autonomic dysreflexia and neuropathic pain (2, 28, 29). Importantly, stretching of these animals did not cause discernible injury to the rodents' hind limb muscles but appeared to impact the spinal neural circuitry (9). Thus, this evidence raises a crucial question—does stretching cause comparable results in humans with SCI? Given the lack of evidence, future research is needed to investigate the impacts of stretching on humans with SCI.

## 4 Discussion

Limited evidence supports stretching as a useful strategy to address spasticity, offering a low-cost, non-invasive option to manage this common impairment. However, some studies suggest that the effects of stretching on joint ROM and contractures are not clinically meaningful, calling into question its widespread application for these purposes. Furthermore, stretching's impact on voluntary motor output and functional recovery is multifaceted and requires further investigation.

The experimental findings in rodent models of SCI subjected to a stretch intervention revealed negative effects on voluntary motor output. Stretching caused impairments in locomotor function, potentially due to sensitization of nociceptor afferents. If this effect translates to humans with SCI, then repeated stretching could sensitize the spinal circuitry, and a relatively small dose of stretching might inhibit voluntary motor output. Inhibition of voluntary motor output can impact the achievement of rehabilitation goals. The possibility of hard-earned gains being reduced by stretching warrants further investigation into the effect of stretching on motor output. Additionally, the role of nociceptive afferents and the observed C-fiber sprouting in spinal neural circuitry highlight the potential interplay between stretching and neural adaptations post-SCI. This, too, warrants further investigation.

Stretching is currently considered to be a desirable intervention for nearly all patients with SCI. However, evaluating relevant research raises questions about its effectiveness and potential for adverse outcomes. In pursuing enhanced motor function and overall well-being following SCI, assessing the use of stretching in SCI rehabilitation paradigms is imperative. Future research should investigate the mechanisms underlying stretch-induced effects and the variables (stretch duration, intensity, variations in approach) that may enhance the beneficial effects of stretching. A comprehensive understanding of stretching, as well as guidelines regarding its appropriate use, are needed to optimize rehabilitation for people with SCI.

## Data Availability

The original contributions presented in the study are included in the article/Supplementary Material, further inquiries can be directed to the corresponding author.
